# ARTnet Perspectives and Contributions to Theranostics

**DOI:** 10.1055/s-0045-1812309

**Published:** 2025-10-14

**Authors:** Roslyn J. Francis, Dale L. Bailey, Kathy Willowson, Melissa J. Latter, Bridget Chappell, George McGill, Michael S. Hofman, Louise Emmett, Andrew M. Scott

**Affiliations:** 1 Department of Nuclear Medicine, Royal Brisbane and Women's Hospital, Brisbane, Australia; 2 Australian Institute for Bioengineering and Nanotechnology, University of Queensland, Brisbane, Australia; 3 Medical School, University of Western Australia and Sir Charles Gairdner Hospital, Perth, Australia; 4 Department of Nuclear Medicine, Royal North Shore Hospital, Sydney, Australia; 5 The Institute of Medical Physics, University of Sydney, Sydney, Australia; 6 Faculty of Medicine and Health at University of Sydney, Sydney, Australia; 7 Department of Molecular Imaging and Therapy at Austin Health, Melbourne, Australia; 8 Department of Molecular Imaging, Princess Alexandra Hospital, Brisbane, Australia; 9 Prostate Cancer Theranostics and Imaging Centre of Excellence (ProsTIC), Molecular Imaging and Therapeutic Nuclear Medicine, Cancer Imaging, Peter MacCallum Centre, Melbourne, Australia; 10 Sir Peter MacCallum Department of Oncology, University of Melbourne, Melbourne, Australia; 11 Department of Nuclear Medicine, St Vincent's Hospital, Sydney and Faculty of Medicine, UNSW Sydney, NSW, Australia; 12 Olivia Newton-John Cancer Research Institute, School of Cancer Medicine at La Trobe University, Melbourne, Australia; 13 Faculty of Medicine, University of Melbourne, Melbourne, Australia

**Keywords:** clinical trials, harmonization, oncology, radiopharmaceuticals, theranostics

## Abstract

Theranostics is a rapidly growing field, providing new therapeutic options for cancer patients. Clinical trials have a key role in establishing the efficacy and safety of new treatments and determining impact on patient care. Multicenter clinical trials with radiopharmaceuticals provide robust data to support clinical implementation; however, there are important considerations to ensure high-quality and reliable clinical data.

Australasian Radiopharmaceutical Trials Network (ARTnet) is a multidisciplinary clinical trials network established in 2014 to facilitate multicenter clinical trials with radiopharmaceuticals in Australia. Over the last decade, ARTnet has supported impactful, prospective clinical trials through quality activities and engagement. In theranostics, Australia has had a key role in clinical translation and generating evidence for safety and efficacy, resulting in regulatory approval and health care funding internationally. This report describes the development of ARTnet as a clinical trials network, highlighting the intent, current status, and operations of ARTnet, with a particular focus on theranostics.

## Introduction

Over the last decade, there has been a rapid growth in novel radiopharmaceuticals for imaging and therapy. Precision medicine approaches, such as theranostics in which imaging with a diagnostic tracer confirms target expression, followed by administration of a therapeutic radiopharmaceutical directed to the target, allow clinicians to “treat what you see.” 1 As a targeted therapy, theranostics applications have been shown to demonstrate efficacy with favorable toxicity profiles. 1

Worldwide, novel radiopharmaceuticals represent a rapidly expanding industry. 1 2 3 The annualized growth rate of the radiopharmaceutical market is high, driven initially by disease-specific positron emission tomography (PET) tracers for imaging, and more recently by therapeutic applications. 2 There are new targets identified, a wide range of targeting molecules from peptides to antibodies, and increasing clinical experience with therapeutic isotopes, including α, β, and auger therapies. 1

In the field of Nuclear Medicine, this is an unprecedented time of growth and opportunity. There are, however, challenges, including ensuring appropriate facilities, expertise, and training to facilitate access and ensure high-quality care in theranostics. Collaboration within Nuclear Medicine and across specialties is required to provide care to the patients who we treat. As new radiopharmaceuticals emerge, the importance of clinical trials is now recognized to be of fundamental importance. There is no longer an acceptance of retrospective single-center studies as providing sufficient evidence for clinical adoption of a new imaging or therapy agent. 4 Instead, over the last decade, there have been an increasing number of prospective clinical trials designed to establish efficacy, safety, cost-effectiveness, and clinical utility of novel radiopharmaceuticals. 1 Evidence generated from these studies has led to regulatory approval, including U.S. Food and Drug Administration (FDA) approval and health care funding of ^177^ Lutetium prostate-specific membrane antigen ([ ^177^ Lu]Lu-PSMA) and [ ^177^ Lu]Lu-DOTA TATE in the United States. 1

Recognizing the importance of multicenter clinical trials with radiopharmaceuticals for imaging or therapy, the Australasian Radiopharmaceutical Trials Network (ARTnet) was launched 10 years ago, to facilitate high-quality clinical trials with radiopharmaceuticals in Australia. The ARTnet model of supporting multicenter clinical trials ensures standardization while reducing duplication at study sites. ARTnet trials utilize a network of sites across Australia to ensure rapid recruitment to clinical trials. Individual sites benefit by gaining experience in theranostics, and clinical teams and their patients have better access to clinical trials. ARTnet has supported several impactful clinical trials in theranostics, with strong engagement in the Nuclear Medicine and research community in Australia. This report outlines the intent, activities, and operations of ARTnet, with the aim of highlighting the importance of collaborative clinical trials in theranostics, leading to high-quality data and impactful outcomes.

## Multicenter Clinical Trials with Radiopharmaceuticals: A Trials Network Approach

ARTnet is a joint venture of Australia's two Nuclear Medicine professional societies, the Australian and New Zealand Society of Nuclear Medicine and Australian Association of Nuclear Medicine Specialists. ARTnet has a mission statement “To promote and facilitate innovative collaborative clinical research utilizing radiopharmaceuticals for imaging or therapy.” ARTnet's Executive Committee is responsible for governance, financial management, and strategic planning. The scientific activities of ARTnet are conducted through the Scientific Committee, which has broad representation across Nuclear Medicine specialists, Nuclear Medicine technologists, physicists, and radiopharmaceutical scientists. 5 ARTnet project officer supports the executive and scientific activities.

**Fig. 1:**
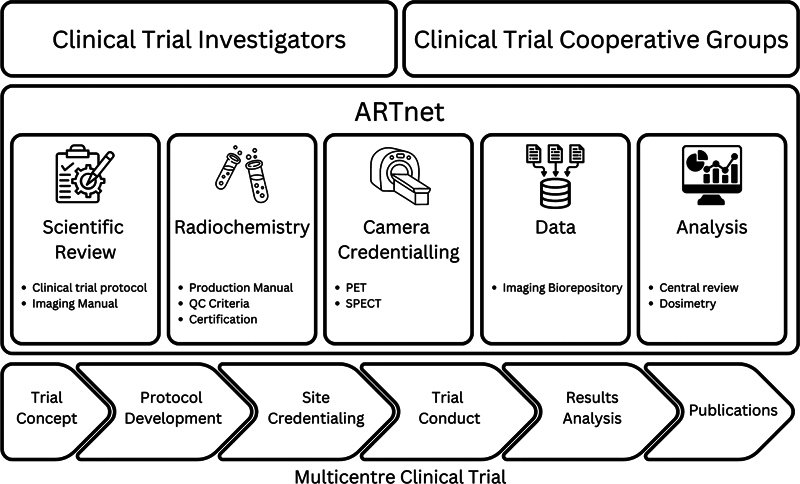
Schematic diagram of ARTnet activities to facilitate multicenter clinical trials with radiopharmaceuticals for imaging or therapy. In collaboration with clinical trial investigators and clinical trial cooperative groups, ARTnet offers several quality activities to support clinical trials, from trial concept and protocol development to credentialing and on-trial activities through to central review and dosimetry analysis. ARTnet, Australasian Radiopharmaceutical Trials Network.

ARTnet conducts several scientific activities in standardization and harmonization of the nuclear medicine components of multicenter clinical trials. This ranges from scientific review, credentialing activities in image acquisition and radiochemistry, centralized imaging data review and an imaging biorepository to support clinical trials. Fig. 1 provides a schematic representation of these activities throughout the conduct of a clinical trial and highlights the important relationship with clinical trial investigators and co-operative trials groups.

The multidisciplinary representation of ARTnet ensures wide expertise available to support study design and implementation. The trials conducted through ARTnet have been predominantly investigator-initiated studies with competitive research grant funding. There is a formalized process for scientific review and endorsement of clinical trial protocols, with a focus on the nuclear medicine aspects of the study. ARTnet also works collaboratively with investigators to develop imaging and radiopharmaceutical manuals. This is particularly important for novel radiopharmaceuticals that do not have established radiochemistry or imaging protocols. Nuclear Medicine technologist expertise supports participant workflow, imaging acquisition protocols, image processing, and upload procedures. Physics representation provides oversight of technical components of imaging, radiation safety, and the camera credentialing program. Development, technology transfer, manufacture, and quality control of radiopharmaceuticals are provided by radiopharmaceutical scientist expertise. Nuclear Medicine specialists contribute to clinical trial design and implementation and support trial conduct and engagement. The broad representation of the ARTnet scientific committee reflects the importance of multidisciplinary expertise and training required for theranostics practice. 6

The credentialing activities of ARTnet have been important for ensuring standardization in clinical trials. The camera validation program utilizes a National Electrical Manufacturers Association (NEMA) NU-2 IEC IQ body phantom, sent to sites with instructions for image acquisition and data upload. The importance of the ARTnet camera validation program was highlighted with gallium-68 PET camera accreditation undertaken for the ProPSMA clinical trial. ProPSMA was a prospective clinical trial at 10 sites, evaluating [ ^68^ Ga]Ga-PSMA-11 for primary staging of prostate cancer. 7 An unexpected 15% underestimation in standardized uptake values (SUV) (range: −23% to −13%) was found in 10/14 PET systems evaluated. 8 This was eventually identified to be due to an incorrect factory-shipped dose calibrator setting. Had the camera validation program not been undertaken, there would have been an unacceptable SUV variation across trial sites for this study.

ARTnet provides a camera validation program for multiple PET radioisotopes, including gallium-68 and fluorine-18, and more recently for longer half-life isotopes such as iodine-124. In theranostic studies, quantitative single-photon emission computed tomography (SPECT) has the potential to be utilized for post-therapy dosimetry estimates and to gain early indication of therapeutic response. 9 10 ARTnet supports SPECT camera validation with lutetium-177 and iodine-131 for theranostic trials. To facilitate the evaluation of long half-life isotopes in PET or SPECT, an ARTnet insert has been developed to overcome the logistical challenges of contamination of the phantom, as the insert can be removed and easily stored after use. 11 This is particularly important as ARTnet NEMA IQ phantoms are transported around Australia to sites for the validation activities.

Validation of PET and SPECT systems is an important quality activity for multicenter clinical trials with theranostics. ARTnet acceptance specification for quantitative accuracy of reconstructed images is within 5% of the true value for PET and within 10% of the true value for SPECT. 11 12 This allows comparability of semiquantitative PET measurements, such as SUV, across sites. This was highlighted in the TheraP trial, in which [ ^68^ Ga]Ga-PSMA-11 PET was undertaken as a screening investigation and participants were included in the trial if they were demonstrated to have a single site of PSMA-avid disease with SUVmax >20, and all sites of measurable disease with PSMA SUV >10. 13 14 To ensure comparability of the semiquantitative parameters across all 11 participating sites in TheraP, standardized imaging protocols, camera acquisition parameters, and camera validation were required.

In Australia, the large geographic distance between study sites is challenging for distribution of radiopharmaceuticals with short half-lives. On-site radiopharmaceutical manufacturing, particularly of short-lived tracers, is undertaken in academic/tertiary hospitals by trained radiopharmaceutical scientists. ARTnet facilitates this process for clinical trials with novel radiopharmaceuticals through standardized processes outlined in production manuals, including synthesis techniques and quality control release criteria. Production methods may include manual, semi-automated, automated, or kits; however, the final product must conform to specification and quality control criteria prior to release. 15 ARTnet provides oversight of credentialing activities, including review of batch records and site visits, to ensure comparability of radiopharmaceuticals for imaging or therapy across sites.

The incorporation of central image review into a multicenter clinical trial design is a quality activity to confirm patient eligibility, and/or to validate imaging outcomes as study endpoints. In ENZAp, TheraP, and UpfrontPSMA theranostic trials, central review was undertaken to confirm the imaging phenotype at screening. This ensured that across all sites, the patient cohort was confirmed to meet the study entry criteria according to predefined imaging parameters. In the I-FIRST clinical trial, ARTnet supports central review of iodine-124 PET and [ ^18^ F]fluorodeoxyglucose (FDG) PET scans, pre- and post-redifferentiation therapy. I-FIRST also incorporates a centralized prospective dosimetry analysis embedded in the trial design, with predetermined criteria for proceeding to radioactive iodine therapy. Centralized review activities can be highly beneficial as local investigators gain clinical experience within a trial, with central review undertaken for consistency and trial quality.

Increasingly, images acquired and collected in clinical trials are recognized as a valuable source of data. Analysis of imaging biomarkers for predictive or prognostic indicators of response in theranostics trials was demonstrated in the TheraP clinical trial. [ ^68^ Ga]Ga-PSMA-11 SUV _mean_ at tumor sites was predictive of response to [ ^177^ Lu]Lu-PSMA therapy, and [ ^18^ F]FDG PET tumor volume was prognostic. 16 An imaging data repository to collect DICOM files from multiple trial sites for review and analysis is therefore a critical component of clinical trials with theranostics. Ideal characteristics of an imaging biorepository include reliable, robust, and secure data collection of anonymized images, with the capability for central review and analysis. For academic studies, this resource should be cost-effective. This is an area of development for ARTnet, and a capability that will add significant support for multicenter clinical trials.

## ARTnet Theranostic Clinical Trials

ARTnet supports both diagnostic and therapeutic clinical trials with radiopharmaceuticals, in oncology and non-oncology applications. Combined, there have been more than 1,500 patients recruited into ARTnet-facilitated trials since 2014, with over 600 patients recruited into theranostic trials ( Table 1 ).

**Table 1:** ARTnet credentialled clinical trials in theranostics demonstrating the number of trial sites and enrolled patients

ARTnet clinical trials—THERANOSTICS	Trial sites	Total patients	Trial status	Refs.
**TheraP** (ANZUP 1603; NCT03392428) A randomized phase 2 trial of ^177^ Lu-PSMA617 theranostic versus carbazitaxel in progressive metastatic castration resistant prostate cancer.	11	200	Completed	13 14
**ENZA-p** (ANZUP 1901; NCT04419402) A randomized phase II trial using PSMA as a therapeutic agent and prognostic indicator in men with metastatic castrate-resistant prostate cancer treated with enzalutamide	15	160	Completed	22 23 26
**UpFrontPSMA** (ANZUP 1904; NCT04343885) A randomized phase 2 study of sequential ^177^ Lu-PSMA617 and docetaxel versus docetaxel in metastatic hormone-naive prostate cancer	11	130	Completed	24 25
**EVOLUTION** (ANZUP 2001; NCT05150236) Phase II study of radionuclide ^177^ Lu-PSMA therapy versus ^177^ Lu-PSMA in combination with Ipilimumab and nivolumab for men with metastatic castration resistant prostate cancer (mCRPC)	9	100	Closed	
**I-FIRST Study** (ONJ2021–006; NCT05182931) A prospective, multi-center trial of TKI redifferentiation therapy in patients with RAIR thyroid cancer	9	38/80	Open	

Abbreviation: ANZUP, New Zealand Urogenital and Prostate Cancer clinical trials group.

Note: Recruitment to I-FIRST is ongoing.

The impact of an Australian clinical trials network in Nuclear Medicine was highlighted by the early clinical trials supported by ARTnet with PSMA PET imaging. A management impact study conducted at four sites in Australia in 431 men with prostate cancer demonstrated that PSMA PET led to a change in planned management in 51% men, with the impact highest in men with biochemical failure (62% change in management intent). 17 The ProPSMA clinical trial established the role of PSMA PET in staging of men with intermediate/high risk prostate cancer, demonstrated improved accuracy, less equivocal findings, higher reporter agreement, reduced radiation dose, and cost effectiveness over conventional imaging. 7 18 This highly impactful study recruited 300 patients from 10 Australian sites, with a recruitment timeline 9 months ahead of schedule, and has led to funding of PSMA PET imaging for men with prostate cancer in Australia and internationally and incorporation into management guidelines for care of men with prostate cancer. 19 20

In theranostics, TheraP was the first prospective clinical trial supported by ARTnet. TheraP was conducted in 11 sites across Australia, with several of the trial sites, at the time of study commencement, having either minimal or no experience with theranostics or specifically with [ ^177^ Lu]Lu-PSMA therapy. This landmark randomized phase II clinical trial demonstrated the efficacy of [ ^177^ Lu]Lu-PSMA-617 with a higher prostate-specific antigen (PSA) 50 response rate than cabazitaxel, with lower side effects. 13 14 ARTnet activities to support TheraP included protocol/imaging manual review, PET camera credentialing, and radiopharmaceutical support for onsite production of [ ^68^ Ga]Ga-PSMA-11 and [ ^177^ Lu]Lu-PSMA-617. Recruitment targets for TheraP were met approximately 4 months ahead of schedule. This prospective clinical trial has resulted in several highly cited publications, 13 14 21 and has contributed to FDA approval of [ ^177^ Lu]Lu-PSMA-617 in United States and to recent Medicare Services Advisory Committee regulatory approval in Australia.

Further prostate cancer theranostic clinical trials facilitated by ARTnet include ENZAp, 22 23 UpfrontPSMA, 24 25 and Evolution. The ENZAp trial, a randomized controlled phase 2 trial evaluating [ ^177^ Lu]Lu-PSMA-617 in addition to enzalutamide in men with high-risk metastatic castration-resistant prostate cancer, was conducted at 15 sites in Australia. Published in 2024, this study demonstrates improvement in PSA progression-free survival utilizing combined enzalutamide and [ ^177^ Lu]Lu-PSMA-617, with the combination treatment being well tolerated. 23 Recently released survival analysis demonstrates an overall survival advantage to men treated with combination enzalutamide and [ ^177^ Lu]Lu-PSMA-617 compared with enzalutamide alone, providing important data on potential biological effects of timing, sequencing, and combination therapies with [ ^177^ Lu]Lu-PSMA-617. 26 This study also includes integrated imaging outcomes into the study design, for which standardization of imaging and radiochemistry for the trial will ensure a valuable dataset for subsequent analysis.

The recently published UpfrontPSMA clinical trial demonstrates the safety and efficacy of [ ^177^ Lu]Lu-PSMA-617, followed by docetaxel chemotherapy in high-volume metastatic hormone-sensitive prostate cancer, compared with docetaxel alone. 25 This study, conducted at 11 sites in Australia, demonstrated a greater proportion of men with undetectable PSA at 1 year, and longer PSA progression-free survival with [ ^177^ Lu]Lu-PSMA-617, followed by docetaxel chemotherapy, compared with docetaxel alone. 25

Lastly, the Evolution clinical trial explores the combination of [ ^177^ Lu]Lu-PSMA-617 theranostic with immunotherapy in castrate-resistant metastatic prostate cancer. Recruitment has been completed for this trial and outcomes are awaited.

I-FIRST clinical trial is a prospective multicenter study across seven sites in Australia, evaluating redifferentiation therapy in radioiodine-refractory thyroid cancer. This study includes quantitative imaging with [ ^18^ F]FDG, iodine-124 PET, and iodine-131 SPECT imaging. Novel approaches to site validation for nonstandard quantitative imaging radiotracers, such as iodine-124 and iodine-131 using the ARTnet insert, have been undertaken for this trial. 12 In addition to facilitating protocol review and site credentialing for PET and SPECT, ARTnet is also supporting the imaging biorepository and central review/dosimetry for the trial, using XNAT capability. Prospective dosimetry is undertaken in the I-FIRST trial using iodine-124 PET to determine the patient cohort proceeding to iodine-131 therapy following redifferentiation. Performing central review and dosimetry, and ensuring PET camera performance through ARTnet credentialing, will ensure comparability across all study sites in Australia. This study has commenced recruitment in 2023, with study outcomes expected by 2026.

## Discussion

Over the last decade, ARTnet, as a Nuclear Medicine trials network, has facilitated multiple highly impactful multicenter trials in theranostics in Australia. The trials have been undertaken as investigator-initiated, research grant–funded studies and have been collaborative across sites, specialties, and expertise. This is highlighted by the strong engagement with other co-operative groups in Australia, including Australian and New Zealand Urogenital and Prostate (ANZUP) Cancer clinical trials group, Trans-Tasman Oncology Group (TROG), Australasian Leukaemia and Lymphoma Group (ALLG), and Cooperative Trials Group for Neuro-Oncology (COGNO). The partnership and engagement with other co-operative trials groups have been highly beneficial for the clinical trials environment in Australia and are key contributing factors to impactful evidence-based trials that are translatable into improved clinical care.

The role of ARTnet has been to enable and support the trials, through standardization and quality activities. Similar programs of site credentialing and camera validation are undertaken by Society of Nuclear Medicine and Molecular Imaging (SNMMI) Clinical Trials Network (CTN) and European Association of Nuclear Medicine (EANM) Research Ltd (EARL); however, ARTnet has extended this to include protocol review, radiochemistry credentialing and central image review. One of the challenges that ARTnet is seeking to address is how to facilitate multinational clinical trials, as this would further enhance recruitment targets and accelerate translation of novel radiopharmaceuticals from discovery to clinical implementation through clinical trials. The mutual acceptance of camera credentialing programs (for example, SNMMI CTN, EARL, and ARTnet) and collaboration in developing standardized processes for camera credentialing would be an important initial step in facilitating international collaboration in clinical trials.

Performing prospective clinical trials is fundamental to generating evidence of efficacy, safety, and economic evaluation (cost–benefit), which are required for regulatory approval and funding. The funding of PSMA PET imaging in Australia was supported by prospective clinical trials demonstrating management impact, 17 diagnostic performance and safety, 7 and economic benefit. 18 As discussed, in theranostics, the TheraP trial has contributed to approvals of [ ^177^ Lu]Lu-PSMA internationally, with the results of ENZAp and UpfrontPSMA likely to further add to the treatment landscape in prostate cancer. In differentiated thyroid cancer, the results of the I-FIRST study will be internationally impactful for patients with radioiodine-refractory disease.

As evidence emerges of the efficacy of theranostics in the management of patients with cancer, there is an increasing importance on training programs and multidisciplinary care. 6 27 Clinical trials provide important opportunities for site engagement and experience in theranostics, strengthen multidisciplinary care, and support training and education in Nuclear Medicine. A comprehensive understanding of the principles of theranostics is important for investigators leading clinical trials with radiopharmaceuticals, especially with the wide range of radioisotopes and targeting molecules now available in the research pipeline.

The ARTnet model of facilitating clinical trials through standardization activities and by providing multidisciplinary expertise to support trial design and conduct could be readily adopted by other countries and regions. Developing a platform and environment for multicenter clinical trials with radiopharmaceuticals would have wide benefits that may include improving research grant funding success, extending expertise in clinical trials beyond single-center sites, contributing to education and training, and ensuring successful clinical trials that meet recruitment targets and are designed and conducted to ensure high-quality data.

As technology changes, and there are advancements in new imaging and radiochemistry techniques, it will be important that ARTnet continues to adapt, ensuring it is contemporary and inclusive in clinical trial opportunities. Funding for clinical trials is challenging, in particular for investigator-initiated/academic trials, with government-funded research grants being highly competitive and resources required to conduct phase II/III clinical trials increasingly expensive. This will remain a challenge for Nuclear Medicine, as we balance the growth in novel radiopharmaceuticals with the requirements of designing and conducting high-quality trials to ensure impactful outcomes that will improve the care and well-being of patients.

## Conclusion

The ARTnet model of facilitation of clinical trials is one that has been particularly suited to Australia with small population and geographic challenges. This model is however one that can be readily adopted by other countries. A multicenter approach to clinical trials enhances recruitment, strengthens local site experience, provides opportunities for patients for clinical trial participation, and provides a robustness to clinical trial data. Multidisciplinary collaboration and expertise are fundamental to ensuring high-quality study design and conduct, leading to impactful clinical trials with meaningful patient outcomes.
